# Demonstration of entanglement-enhanced phase estimation in solid

**DOI:** 10.1038/ncomms7726

**Published:** 2015-04-02

**Authors:** Gang-Qin Liu, Yu-Ran Zhang, Yan-Chun Chang, Jie-Dong Yue, Heng Fan, Xin-Yu Pan

**Affiliations:** 1 Beijing National Laboratory for Condensed Matter Physics, Institute of Physics, Chinese Academy of Sciences, Beijing 100190, China; 2 Collaborative Innovation Center of Quantum Matter, Beijing 100190, China

## Abstract

Precise parameter estimation plays a central role in science and technology. The statistical error in estimation can be decreased by repeating measurement, leading to that the resultant uncertainty of the estimated parameter is proportional to the square root of the number of repetitions in accordance with the central limit theorem. Quantum parameter estimation, an emerging field of quantum technology, aims to use quantum resources to yield higher statistical precision than classical approaches. Here we report the first room-temperature implementation of entanglement-enhanced phase estimation in a solid-state system: the nitrogen-vacancy centre in pure diamond. We demonstrate a super-resolving phase measurement with two entangled qubits of different physical realizations: an nitrogen-vacancy centre electron spin and a proximal ^13^C nuclear spin. The experimental data shows clearly the uncertainty reduction when entanglement resource is used, confirming the theoretical expectation. Our results represent an elemental demonstration of enhancement of quantum metrology against classical procedure.

## Abstract (web summary)

Quantum parameter estimation aims to improve on classical statistical precision where uncertainty decreases proportionally with the square root of the repetition number. Here, Liu *et al.* demonstrate entanglement-enhanced phase estimation at room-temperature in a nitrogen-vacancy centre in pure diamond.

## Graphical abstract

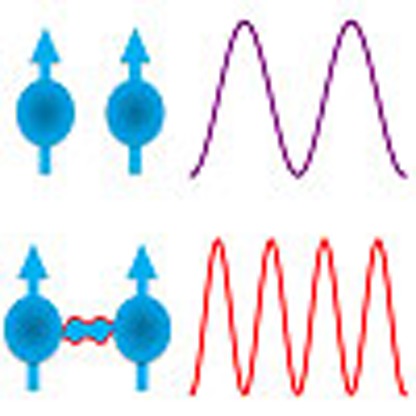

Information about the world is acquired by observation and measurement, the results of which are subject to error^1^. The classical approach to reduce the statistical error is to increase the number of resources for the measurement in accordance with the central limit theorem; however, this method sometimes seems undesirable and inefficient^2^. Quantum parameter estimation, the emerging field of quantum technology, aims to yield higher statistical precision of unknown parameters by harnessing entanglement and other quantum resources than purely classical approaches^3^. Since this quantum-enhanced measurement will benefit all quantitative science and technology, it has attracted a lot of attention as well as contention. Using *N* independent particles to estimate a parameter *ϕ* can achieve at best the standard quantum limit (SQL) or called shot-noise limit scaling as 
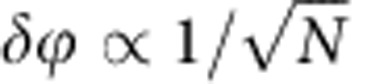
 while it is believed that using *N* entangled particles and exotic states such as NOON states in principle is able to achieve the inviolable Heisenberg limit scaling as *δϕ*∝1/*N* (refs 4, 5). In such circumstances, there are many efforts using non-classical states and quantum strategy for sub-SQL phase estimation in different physical realizations, such as optical interferometry 2,6,7,8,9, atomic systems^10^,^11^ and Bose-Einstein condensates^12^,^13^.

In this paper, we report the first room-temperature proof-of-principle implementation of entanglement-enhanced phase estimation in a solid-state system: the nitrogen-vacancy (NV) centre in pure diamond single crystal. An individual NV center can be viewed as a basic unit of a quantum computer in which the nuclear spin with a long coherence time performs as the memory and the centre electron spin with a high control speed acts as the probe. This solid-state system is one of the most promising candidates for quantum information processing (QIP), and many coherent control and manipulation processes have been performed with this system^14^,^15^,^16^,^17^,^18^,^19^,^20^,^21^,^22^,^23^,^24^,^25^,^26^,^27^,^28^,^29^. Here we demonstrate a super-resolving phase measurement with two entangled qubits of different physical realizations: a NV centre electron spin and a proximal ^13^C nuclear spin. We are able to improve the phase sensitivity by factors close to 
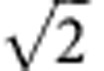
 compared with the classical scheme, which conforms to the fundamental Heisenberg limit. As we have entangled two qubits with different physical realizations, our results represent a more generalized and elemental demonstration of enhancement of quantum metrology. Moreover, our system has overcomed the defects of post-selection in the most common optical systems which are fatal due to the fact that the measurement trials abandoned will eliminate the quantum advantage over classical strategy.

## Results

### System description

The phase estimation scheme is implemented by optically detected magnetic resonance (ODMR)^14^,^16^ technique on a home-built confocal microscope system. The description of the system can be found in ref. 27. The spin-1 electron spin of NV centre has triplet ground states with a zero-field splitting of Δ≈2.87 GHz between the states |0› and |±1›. As an external magnetic field of about 507 Gauss is applied along [111] direction of the diamond crystal, the degeneration of |±1› states can be well relieved, and the first qubit is encoded on the |0› and |−1› subspace. The electron spin state can be initialized to |0› state by a short 532 nm laser pulse (3 *μ*s) and manipulated by resonant microwave (MW) pulses of tunable duration and phase. The electron spin state is readout by collecting the spin-dependent fluorescence. To enhance the fluorescence collection efficiency, a solid immersion lens (SIL)^30^ is etched above the selected NV center, typical count rate in this experiment is 250 k.p.s. with SIL, see Methods for details.

**Figure 1:**
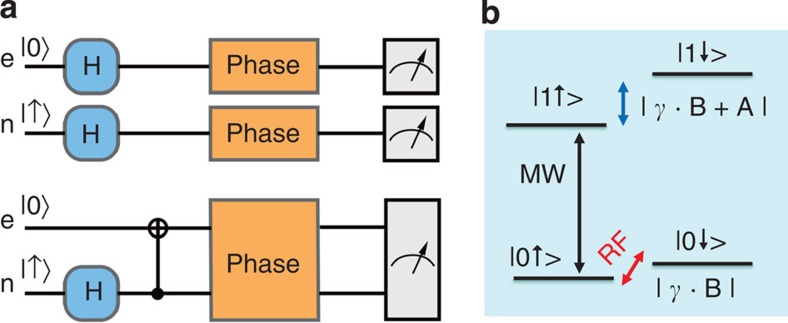
General scheme and system description. (**a**) Phase estimation schemes of the independent states and the electron–nuclear-entangled state. By harnessing entanglement, quantum metrology yields higher statistical precision than classical approaches. (**b**) Energy levels and physical encoding of the two-bit system. The electron spin and a nearby ^13^C nuclear spin of an NV center are employed to demonstrate the metrology scheme. At excited-state level anti-crossing (ESLAC), both spins can be polarized, manipulated and readout with high fidelity. Two-bit conditional quantum gates are implemented by applying selective microwave (MW) or (RF) pulses.

The second qubit is encoded on the |↑› and |↓› states of a nearby ^13^C nuclear spin. See Fig. 1b for the energy levels of the two-qubit system. The coupling strength between the target nuclear spin and centre electron spin is 12.8 MHz, which indicates the ^13^C atom sites on the third shell from the NV centre^31^. The polarization and readout procedure of the nuclear spin is more complicated than that of a electron spin. The 507 Gauss magnetic field causes excited-state level anti-crossing (ESLAC) of centre electron spin, in which the optical spin polarization of centre electron will transfer to nearby nuclear spins^32^,^33^. So the host ^14^N nuclear spin, the nearby ^13^C nuclear spin as well as the center electron spin are polarized by the same laser pulse under this magnetic field. To readout the nuclear spin state, a mapping gate, which transfers nuclear spin state to electron spin, and a following optical readout of electron spin state are employed^17^,^33^, see Methods for details.

The nearby nuclear spin couples to the centre electron spin through strong dipolar interaction, which provides excellent conditions to implement two-qubit controlled gate. On the one hand, the resonant frequency of |0↑›↔|1↑› transition and |0↓›↔|1↓› transition are separated by 12.8 MHz from each other, so we can selectively manipulation one branch of nuclear spin with high fidelity while keep the other branch untouched (using weak MW pulses, see black arrow in Fig. 1b). On the other hand, the nuclear spin state evolution is strong affected by the state of electron spin: when electron spin is on the |0› state (or |−1› state), the dynamics of the nuclear spin is dominated by the external magnetic filed (or the dipolar interaction, respectively), its Zeeman splitting between the |↑› and |↓› states is about 500 kHz, which is far away from the dipolar interaction strength of 12.8 MHz. Therefore, we can selectively manipulate nuclear spin state in one branch of electron spin, as well (using RF pulses, see red arrow in Fig. 1b and Supplementary Note 1).

### Phase preparation and measurement

**Figure 2:**
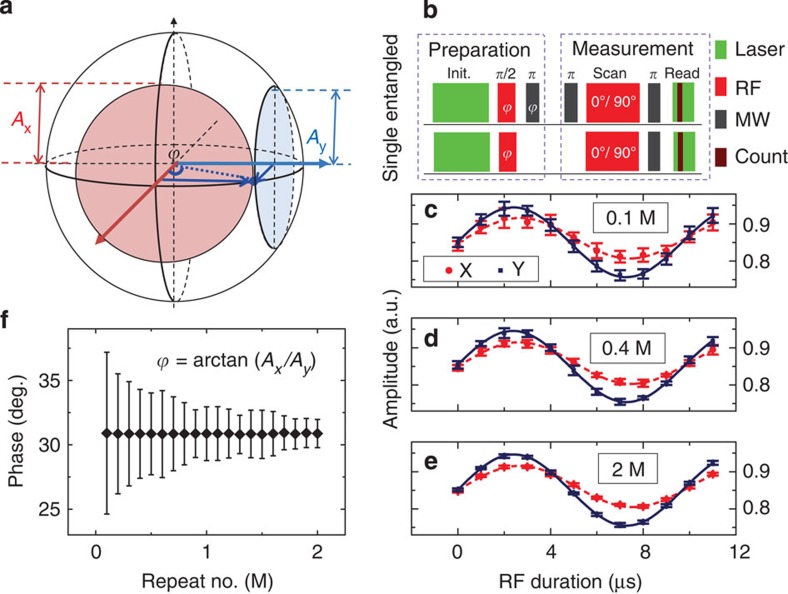
Phase preparation and measurement. (**a**) For each unknown state sitting on the equatorial plane of the Bloch sphere, we compare the amplitudes of the two Rabi signals from this state (under orthogonal microwave pulses driven) with extract the original phase information: *ϕ*=arctan (*A*_*x*_/*A*_*y*_). (**b**) Upper pane: pulse sequence to prepare and measure electron–nuclear spin entanglement state 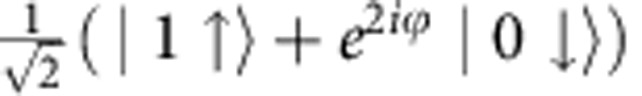. Lower panel: pulse sequence to prepare and measure nuclear spin superposition state 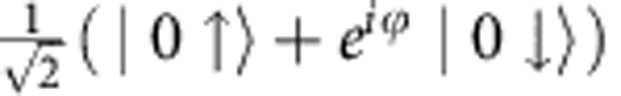. (**c**–**e**) Nuclear Rabi signals for phase measurement, with 0.1, 0.4 and 2 M repetition of pulse sequence in (**b**). The input phase *ϕ* is 30°. Solid circle with blue fitting line is driven by 0° RF pulse (X measurement), and square with red dash fitting line is driven by 90° RF pulse (Y measurement). (**f**) Dependence of measured phase and its standard deviation on repeat number.

Figure 2b describes the pulse sequence to prepare and measure the phase of a superposition state. Take nuclear spin for example, the qubit is defined in a rotating frame with frequency equalling to the energy splitting between |0↑› and |0↓› states. After polarized to |0↑› by laser pulse, a resonant RF π/2 pulse brings the system to 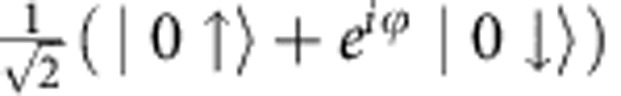 state. The phase of this state is determined by the relative phase of the applied RF pulse, which is tunable in experiment. The phase of electron superposition state is prepared in the same way, with resonant MW pulses.

The phase information of a superposition state is detected by converting it to population information of the spin qubits and a following optical readout. To eliminate the system error in long time measurement, we use a self-calibration measurement scheme as shown in Fig. 2a. For each unknown state siting on the equatorial plane of the Bloch sphere, we measure the Rabi oscillations driven by two orthogonal MW pulses (0° and 90°), and compare the amplitudes of the two Rabi signal to extract the original phase information: 
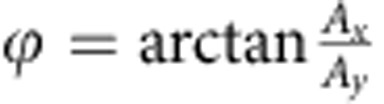
, where *A*_*x*_ and *A*_*y*_ are the amplitudes of Rabi signals driven by 0° and 90° MW pulses, respectively. Note that this is a single-spin experiment, and we need to repeat the pulse sequence many times to get a reliable signal-to-noise ratio. Fig. 2c–e presents the nuclear Rabi signal of 0.1, 0.4 and 2 M repeat of the pulse sequence in Fig. 2b, with an input phase of 30°. The error bar of the data point represent s.d. of 10 repeat measurements. It is clear to see from Fig. 2c–e that the signal-to-noise ratio is better as the measurement sequence is repeated more times. In Fig. 2f, the decrease of phase estimation error can be well described by central limit theorem.

**Figure 3:**
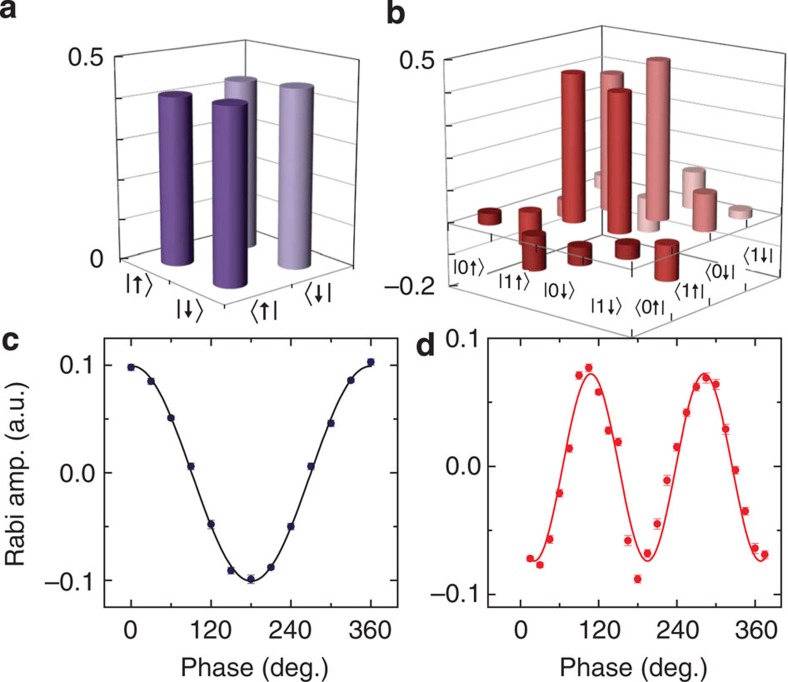
State tomography and phase relation. (**a**) The state tomography result of a nuclear spin superposition state 
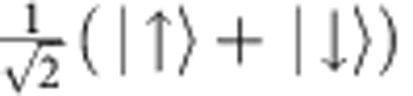
. (**b**) State tomography result of the electron–nuclear-entangled state 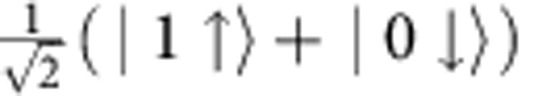. (**c**) Phase relation of an independent state. (**d**) Phase relation of an entangled state. Compared with independent state, the phase relation of entangled state has double frequency dependence on input phase, so a more precise phase estimation result can be achieved with entangled state.

To improve phase estimation accuracy, one can increase the repeat number, which means longer measurement time is needed. An equivalent way is to employ more qubits. As mentioned before, the state of the multiqubit system, independent or entanglement, determines the accuracy limit of phase estimation. For the investigated two-qubit system, the electron and nuclear spin can be prepared and measured independently. Fig. 3a plots the state tomography result of a nuclear spin superposition state. Using such independent state (either nuclear spin or electron spin) will get a phase relation as depicted in Fig. 3c, the amplitude of Rabi signal has cosine dependence on the phase of input state.

The electron and nuclear spin can be prepared in entangled state by combination of MW and RF pulses. As shown in the upper pane of Fig. 2b, after the first RF π/2 pulse (with phase *ϕ*) brings the system to 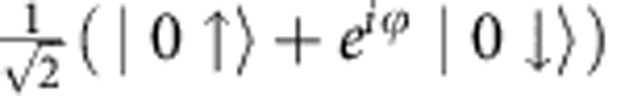 state, a selective MW *π* pulse of |0↑›↔|1↑› transition, which has relative phase *ϕ* to the first RF pulse, brings the system to 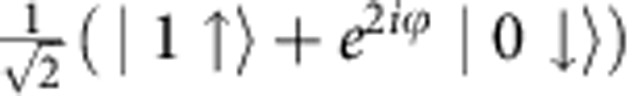 state. The MW and RF channels are synchronized to the same clock reference and relative phase between them is calibrated before each measurement. Typical state tomography result of an electron–nuclear entangled state (*ϕ*=0°) is depicted in Fig. 3b. It is worth noting that the dephasing time of electron spin (0.7 μs, see Methods) is very short compared with the typical manipulation time (for example, 10 μs for a flip operation) of nuclear spin, which limits the QIP applications of this entangled state^22^,^27^,^33^. However, in our phase estimation application, the sensitive phase information is converted to population right after its generation and then only limited by *T*_1_ of electron spin, which is about 5 ms. Meanwhile, the coherence of electron is less affected under MW driving (see Methods), thus the phase of the entangled state is well preserved during the preparation and measurement. As shown in Fig. 3d, the phase relation of the entangled state has double frequency dependence on the phase of input state, so the phase estimation using the entangled state of two-qubit is more precise than that of using two state from independent single qubit.

### Entanglement-enhanced phase estimation

**Figure 4:**
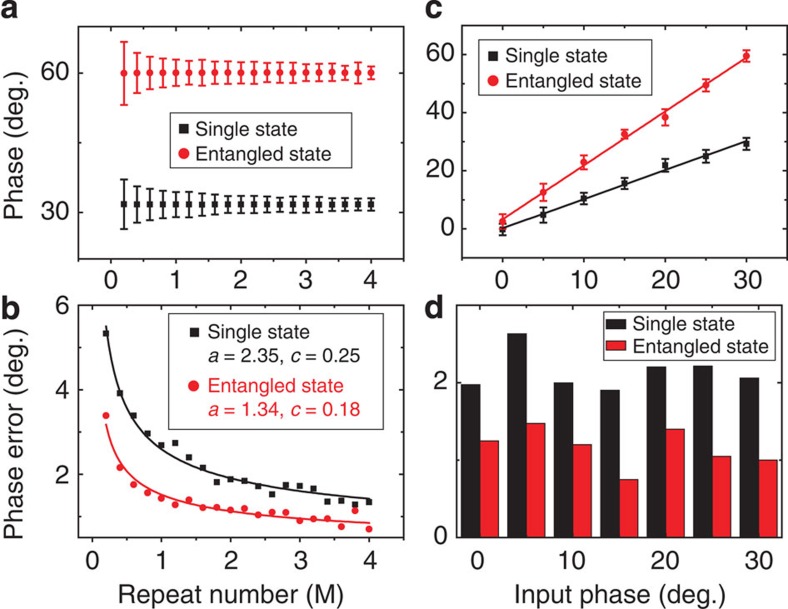
Dependence of phase error on repeat number and input phases. (**a**) Standard deviations of the entangled state and independent state against repeat number. Single-spin states (electron and nuclear) of the same input phase *ϕ*=30° are prepared and measured independently, the phase extracted from each entangled state ‘single measurement’ is 2*ϕ*=60°. (**b**) The phase uncertainty is fitted by function 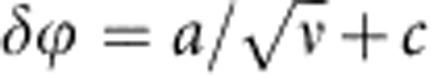 for both single-spin state and the entangled state, where *v* corresponds to repeat number in unit of million (M). The curves show that the phase uncertainty, phase error represented by *δϕ*, of entanglement case is apparently lower than the case of single-spin state. (**c**) The phase estimation results of different input phases including 0°, 5°, 10°, 15°, 20°, 25° and 30°, the repeat number is fixed to 1 M. (**d**) The phase error of different input phases, the repeat number is fixed to 1 M.

To demonstrate the merits of entangled state over independent state in phase estimation application, we compare their performances on different repeat number and different input phase, the measured results are summarized in Fig. 4. The experimental procedure is: first, single-spin states (electron and nuclear) of the same input phase (30°) are prepared and measured independently. Then the output phases extracted from the same repeat number are counted together, no weight is added for either electron or nuclear spin states. For a fair comparison with entangled state, half of the statistic samples (*v*) are extracted from electron spin states, and the other half (*v*) are extracted from nuclear spin states. In the case of entangled state phase estimation, the entangled states are prepared and measured using the same repeat number (*v*). Note the same MW and RF channels are used to prepare the independent and entangled states.

As shown in Fig. 4a,c, the phase extracted from each entangled state measurement is 2*ϕ*, so the phase error of input phase is just half of the standard deviation from sample statistic (*δϕ*/2). For the independent-state input, the double sample number (2) only suppresses phase error to 
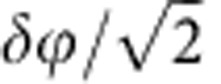
 level, which is larger than entanglement-state input.

Explicitly, we would expect that the phase uncertainty *δϕ* proportion, respectively, to 
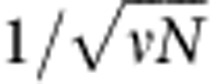
 and 
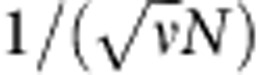
 for single-spin state and the entangled state with *N* being 2 in our experiment since two-qubit entanglement between electron spin and nuclear spin is used as the quantum resource. In Fig. 4b, we consider that identical measurement is repeated *v* times, then a general formulae, 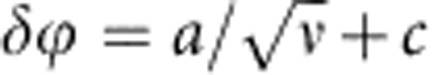, is used to fit experimental data, where *c* is assumed to be a systematic error depending on specific experimental setup. The parameter *a* for scheme of entangled state should be smaller than that of the single-spin scheme, corresponding to smaller uncertainty about the phase, if we assume that the single-spin state and the entangled state are realized by the same physical state. Fig. 4b of the experimental data demonstrates clearly that the precision of phase estimation is enhanced by using entanglement which agrees well with theoretical expectation. Here the discrepancy between theory and experiment is possibly due to two related reasons: the electron spin state and the nuclear spin state are not the same, in particular for their decoherence time, while their similarity is assumed theoretically; the readout of NV centre system can only be by intensity of florescence of the electron spin. Besides the data processing method presented here, we have also tried linear fitting in the log–log scale for s.d. as well as variance (see Supplementary Note 2 and Supplementary Fig. 3 for detail). The obtained results are in good agreement with the results in Fig. 4b, which confirms the validity of our conclusion.

Figure 4c,d show the phase estimation results of different input phases. The phase error of entangled state is smaller than independent state in all input phases, which indicates the enhancement of phase estimation accuracy by entanglement is phase independent.

## Discussion

As summarized in Fig. 4 by different figures of merit quantifying the uncertainty of phase estimation, the entanglement-enhanced precision is clearly shown by experimental data. This experiment demonstrates the advantage of the quantum metrology scheme. Practically by using quantum metrology, the measured physical quantity should have the same interaction on the probe system no matter it is prepared as a single-qubit or entangled state. In our special designed experiment, the measured phases are artificially encoded to the probe state such that the enhancement of precision can be shown by entangled probe state. However, in principle, the confirmation of theoretical expectation by experimental data provides a solid evidence that quantum phase estimation is applicable in this solid-state system.

In this experiment, we use repeating measurement to overcome the low photon collection efficiency of NV center. The phase estimation accuracy can be further improved by employing single-shot measurement technique, which is now available in NV system^19^,^20^,^34^. Although the photon collection efficiency is not perfect (<20%, not every measurement is stored and counted), the following two facts guarantee the reliability of the demonstration: (1) we use the same scheme to measure single and entangled states, that is, the phase information is finally converted to fluorescence signal of NV center and detected. (2) The detection efficiency of the system is stable (though not perfect as single-shot readout) for all the measurement, so we can directly compare the measured phase noise of single and entangled states.

As the phase estimation accuracy is determined by the total number of entangled qubits, a straightforward way to improve the phase accuracy is increasing the involved spin number. The large amount of weakly coupled ^13^C nuclear spins around NV center are one of the best candidates. With the assistance of dynamical decoupling on center electron spin, up to six ^13^C nuclear spins can be coherent manipulated^35^,^36^,^37^. Multiqubit application such as error correction has been demonstrated in this system^24^,^23^.

In conclusion, we report the first room-temperature implementation of entanglement-enhanced phase estimation in a solid-state system: the NV centre in pure diamond. We demonstrate a super-resolving phase measurement with two entangled qubits of different physical realizations: a NV centre electron spin and a proximal ^13^C nuclear spin. Thus, our results represent a more generalized and elemental demonstration of enhancement of quantum metrology against classical procedure, which fully exploits the quantum nature of the system and probes.

## Methods

### Cramér-Rao bound and quantum Fisher information

In the simplest version of the typical quantum parameter estimation problem, we aim to recover the value of a unknown continuous parameter (say phase *ϕ* in Fig. 1a) encoded in a fixed set of states *ρ*_*ϕ*_ of a quantum system^3^. We can obtain a single result ξ via performing a measurement on the system and it is useful to express the measurement in terms of set of POVM {*Ê*_*ξ*_}. With large number of measurements, it is possible to calculate the estimator 
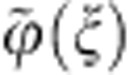
 with the observation conditional probability density function of result ξ given the true values *ϕ*: *p*(*ξ*|*ϕ*)=Tr(*Ê*_*ξ*_ *ρ*_*ϕ*_). When the number of measurements *v* is sufficiently large and the estimation is unbiased^38^,^39^, the root-mean square error for the statistical uncertainty can be shown to obey the well-known Cramér–Rao bound^40^ given by


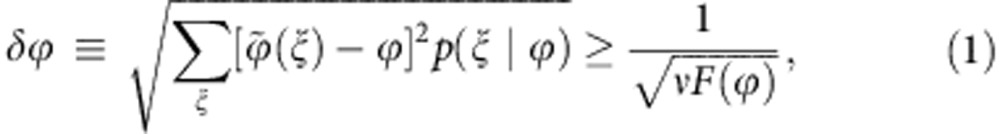


where *F*(*ϕ*)≡∑_*ξ*_ *p*(*ξ*|*ϕ*) [∂_*ϕ*_ ln *p*(*ξ*|*ϕ*)]^2^ is the Fisher information corresponding to the selected POVM and the conditional probability density function of the result. Equation (1) provides a lower bound for the achievable lower bound by choosing the optimal measurement expressed by some POVM 
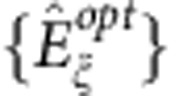
 that maximizes the Fisher information: 
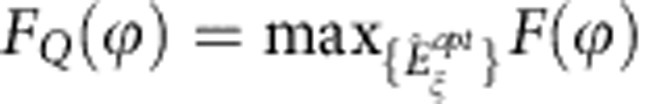
, which is known as the quantum Fisher information^41^.

For the classical scheme with separable probe state 
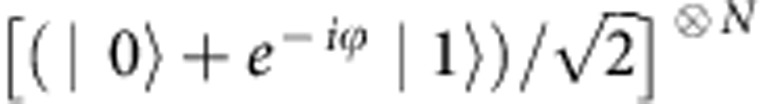
, the lower bound at best leads to the SQL 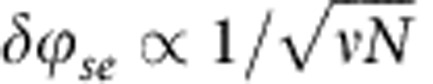. To implement the quantum counterpart of the Heisenberg limit 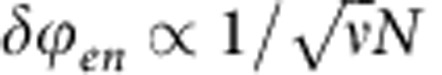, we can choose the GHZ state 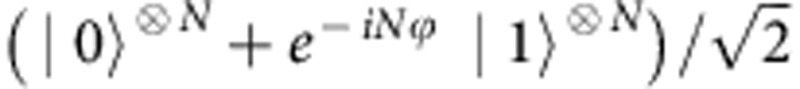 as the optimal probe state. Consider that the qubit number *N*=2, the two-qubit maximally entangled state will obtain a 
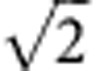
 advantage against the separable state.

### Sample preparation

**Figure 5:**
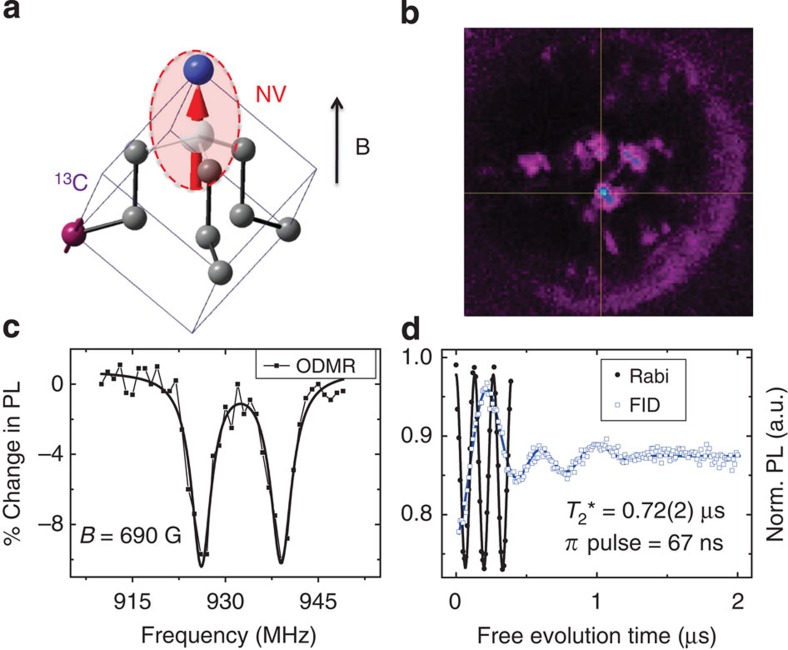
Characterization of the two-qubit system. (**a**) NV centre with a nearby ^13^C nuclear spin. (**b**) 2D fluorescence scan of the FIB-etched solid immersion lens (SIL, 12 *μ*m diameter). The bright spot is the investigated NV centre. (**c**) ODMR of NV electron spin under magnetic field of 690 Gauss. The splitting is caused by the nearby ^13^C nuclear spin. (**d**) Rabi oscillation and free-induction decay (FID) of electron spin (*B*=507 Gauss). Due to the thermal fluctuation of the spin bath, centre electron spin picks up random phase during free precession and loses coherence. With the help of resonant MW pulses, electron spin can be flipped in short time, and is less affected by the bath noise.

High purity single crystal diamond (Element Six, N concentration <5 p.p.b.) is used for this experiment. There is almost no natural NV center in this diamond. NV centres are produced by electron implantation (7.5 Mev) and a following 2 h vacuum annealing (at 800 °C). Due to the random distribution of ^13^C nuclear spins, the spin bath of individual NV center can be very different^31^. We choose NV centers with nearby ^13^C nuclear spins, which can be identified by the extra splitting in ODMR signal, to implement the two-bit metrology scheme. Fig. 5a presents the physical structure of an NV center and a nearby ^13^C nuclear spin. Fig. 5c is ODMR signal of this two-bit system. The coupling strength between electron spin and the selected ^13^C nuclear is 12.8 MHz. Fig. 5b shows two dimensional fluorescence image of the FIB-etched SIL. The cross-cursor marked bright spot (blue) is the one used for this experiment.

### Coherence of electron spin and nearby nuclear spin

In this pure diamond, the coherence of NV electron spin is dominated by the randomly distributed ^13^C nuclear spins (natural abundance, 1.1%). The dephasing time of individual NV centres can be significantly different^42^, from less than 1 *μ*s to nearly 10 *μ*s. From the free-induction decay signal of this NV center in Fig. 5d, we extract the dephasing time 
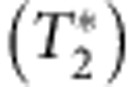
 of this electron spin, which is 0.72 *μ*s and much larger than the time consumption of single manipulation on it (about 70 ns, see Rabi oscillation of electron spin in the same figure). It is worth noting that the dephasing time is not the direct limitation of electron manipulation duration. The latter is usually named *T*_1*ρ*_ and can be characterized by the envelop decay time of electron spin Rabi oscillation^43^. The dephasing time of nuclear spin 
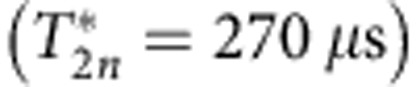
 is much longer than that of electron spin. Meanwhile, the used half *π* pulse of nuclear spin is only several microseconds, so the dephasing effect of nuclear spin can be ignored, See Supplementary Fig. 1 for the FID signal of the nearby nuclear spin.

### Coherent manipulation of electron spin and nuclear spin at ESLAC

**Figure 6:**
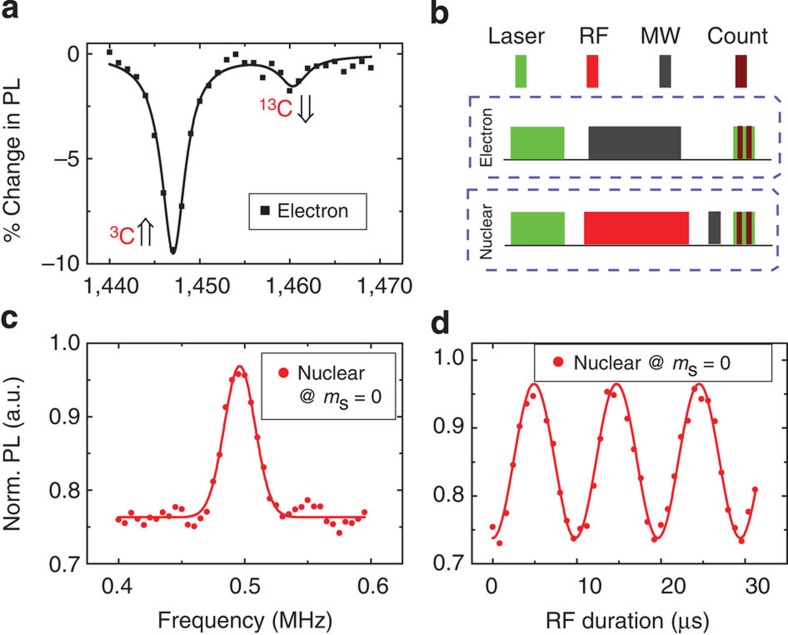
Coherent manipulation of electron spin and nuclear spin at ESLAC. (**a**) ODMR spectrum of electron spin at *B*=507 Gauss. (**b**) Pulse sequence to manipulate electron and nuclear spins at ESLAC. (**c**) ODMR spectrum and (**d**) Rabi oscillation of the nearby ^13^C nuclear spin. At ESLAC, electron spin and nearby nuclear spins (including host ^14^N nuclear spin and nearby ^13^C nuclear spins) can be polarized by a short laser pulse. Then spin states are manipulated by resonant MW or RF pulses. Electron spin states are readout by counting the fluorescence intensity of NV centre; nuclear spin states are mapped to electron spin and readout in the same way. The resonant frequency of this nuclear spin is 495 kHz, which is slightly modified by centre electron spin.

As mentioned in the main text, we work at the ESLAC point to achieve fast and high fidelity initialization of the electron–nuclear two-qubit system. Under an external magnetic field of 507 Gauss (along the quantization axis of the selected NV) and laser excitation (532 nm), the electron and nuclear spins are polarized simultaneously. Fig. 6a shows ODMR spectrum of centre electron spin at such magnetic field. From the contrast difference of two peaks, which correspond to |↑› and |↓› states of ^13^C nuclear spin, we estimate the polarization rate of this nuclear spin is about 85% (in |↑› state). Furthermore, by measuring the pulse-ODMR spectrum of electron spin, we conclude that the host ^14^N nuclear spin is completely polarized under this magnetic field.

Figure 6b shows the pulse sequence of electron and nuclear spin manipulation. After polarization with high fidelity, both spin states can be manipulated with resonant MW (or RF) pulses. For electron spin, the final state is readout by counting the fluorescence intensity of NV center, since |0› state is brighter than |1› state. For nuclear spin state, we use a mapping gate, which is composed by a weak pulse of |0↑›↔|1↑› transition, to transfer the its state to electron spin and then readout optically. For example, an unknown nuclear spin state of |0›⊗(*α*|↑›+*β*|↓›) is transferred to *α*|1↑›+*β*|0↓› after applying the mapping gate. We carefully tuned the MW power and pulse duration to maximum the flip efficiency while avoiding the unwanted non-resonant excitation. By comparing the Rabi amplitude of nuclear spin (Fig. 6d, with mapping gate) and electron spin (Fig. 5d, without mapping gate), we conclude that the mapping gate has transfer efficiency of more than 92%.

Figure 6c,d present the pulse-ODMR spectrum and Rabi oscillation of the nearby nuclear spin when electron spin is at |0› state. The resonant frequency of this nuclear spin is 495 kHz, which is smaller than the Larmor frequency of ^13^C nuclear spin under this magnetic field (542 kHz). We attribute this modification to the ‘enhance effect’ of center electron spin. As the nuclear spin is close to the electron spin, the nonsecular terms of their dipole interaction contribute some electronic character to the nuclear-spin levels and modify its magnetic moment^16^. The Rabi frequency of nuclear spin is about 100 kHz, which reaches 20% of the Zeeman splitting, such fast manipulation also benefits from the electron enhance effect. We discuss the validity of rotating wave approximation in Supplementary Note 1.

### Synchronization of pulse generators and phase calibration

Synchronization of the MW and RF generators is one of the main challenges in this experiment. We use the same clock reference for all the generators. For each cable connection and pulse sequence, we measure the phase of prepared state as we scan the phase of input MW pulses. This gives us a phase relation between MW and RF channels, which is used to compensate the difference between the two rotating frames. We check the phase relation before and after each data acquisition. The phase drift of our system is about 2° in 2 h measurement.

### Data normalization and state tomography

Since the population information of electron spin is the only directly measurable signal in NV system, we normalize all the data to the fluorescence intensity of electron spin |0› state. Specifically, we apply two readout pulses (300 ns) at the end of each measurement. See pulse sequences in Figs 2b and 6b. The first readout pulse gets the instant population information of NV electron spin, and the second readout pulse (1 μs later) records a reference for the first one, as electron spin is polarized to |0› again after the 1-μs laser excitation. The ratio between the first signal and the reference signal is used for further data analysis, such as phase estimation or state tomography.

To carry out state tomography, we adopt the method detailed in refs 18, 27. Total three working transitions, |0↑›↔|0↑›↔|1↑› and |0↓›↔|1↓› are selected. The real and imaginary parts of the matrix elements in each working transition are measured by using RF (or MW) pulses of 0° and 90° phases, respectively. Other three transitions are measured in the same way, but extra transfer pulses are added before Rabi measurement in the working transitions. The full procedure of state tomography can be found in Supplementary information (Supplementary Fig. 2).

## 

## Author contributions

X.-Y.P. and H.F. designed the experiment. X.-Y.P. is in charge of the experiment, H.F. is in charge of the theory. G.-Q.L., Y.-C. C. and X.-Y.P. performed the experiment. Y.-R. Z. and J.-D. Y carried out the theoretical study. G.-Q.L. and Y.-R. Z. wrote the paper. All authors analysed the data and commented on the manuscript.

## Additional information

**How to cite this article:** Liu, G.-Q. *et al.* Demonstration of entanglement-enhanced phase estimation in solid. *Nat. Commun.* 6:6726 doi: 10.1038/ncomms7726 (2015).

## Supplementary Material

Supplementary Information Supplementary Figures 1-3, Supplementary Notes 1-2 and Supplementary References

## References

[b1] Helstron C. W. Quantum detection and estimation theory Academic Press (1976) .

[b2] Israel Y., Rosen S. & Silberberg Y. Supersensitive polarization microscopy using NOON states of light. Phys. Rev. Lett. 112, 103604 (2014) . 24679294 10.1103/PhysRevLett.112.103604

[b3] Giovannetti V., Lloyd S. & Maccone L. Advances in quanum metrology. Nat. Photon. 5, 222–229 (2011) .

[b4] Giovannetti V., Lloyd S. & Maccone L. Quantum metrology. Phys. Rev. Lett. 96, 010401 (2006) . 16486424 10.1103/PhysRevLett.96.010401

[b5] Giovannetti V. & Maccone L. Sub-Heisenberg estiamtion strategies are ineffective. Phys. Rev. Lett. 108, 210404 (2012) . 23003224 10.1103/PhysRevLett.108.210404

[b6] Nagata T., Okamoto R., O’Brien J. L., Sasaki K. & Takeuchi S. Beating the standard quantum limit with four-entangled photons. Science 316, 726–729 (2007) . 17478715 10.1126/science.1138007

[b7] Afek I., Ambar O. & Silberberg Y. High-NOON quantum and classical light states by mixing quantum and classical light. Science 328, 879–881 (2010) . 20466927 10.1126/science.1188172

[b8] Xiang G. Y., Higgins B. L., Berry D. W., Wiseman H. M. & Pryde G. J. Entanglement-enhanced measurement of a completely unknown optical phase. Nat. Photon. 5, 43–47 (2011) .

[b9] Higgins B. L., Berry D. W., Barlett S. D., Wiseman H. M. & Pryde G. J. Entanglement-free Heisenberg-limited phase estimation. Nature 450, 393–396 (2007) . 18004379 10.1038/nature06257

[b10] Meyer V. *et al.* Experimental demonstration of entanglement-enhanced rotation angle estimation using trapped ions. Phys. Rev. Lett. 86, 5870 (2001) . 11415382 10.1103/PhysRevLett.86.5870

[b11] Schleier-Smith M., Leroux H. I. D. & Vuletić V. States of an ensemble of two-level atoms with reduced quantum uncertainty. Phys. Rev. Lett. 104, 073604 (2010) . 20366883 10.1103/PhysRevLett.104.073604

[b12] Jo G. B. *et al.* Long phase coherence time and number squeezing of two Bose-Einstein condensates on an atom chip. Phys. Rev. Lett. 98, 030407 (2007) . 17358668 10.1103/PhysRevLett.98.030407

[b13] Sørensen A., Duan L. M., Cirac J. I. & Zoller P. Many-particle entanglement with Bose-Einstein condensates. Nature 409, 63–66 (2001) . 11343111 10.1038/35051038

[b14] Gruber A. *et al.* Scanning confocal optical microscopy and magnetic resonance on single defect centers. Science 276, 2012–2014 (1997) .

[b15] Jelezko F. *et al.* Observation of coherent oscillation of a single nuclear spin and realization of a two-qubit conditional quantum gate. Phys. Rev. Lett. 93, 130501 (2004) . 15524692 10.1103/PhysRevLett.93.130501

[b16] Childress L. *et al.* Coherent dynamics of coupled electron and nuclear spin qubits in diamond. Science 314, 281–285 (2006) . 16973839 10.1126/science.1131871

[b17] Gurudev Dutt M. V. *et al.* Quantum register based on individual electronic and nuclear spin qubits in diamond. Science 316, 1312–1316 (2007) . 17540898 10.1126/science.1139831

[b18] Neumann P. *et al.* Multipartite entanglement among single spins in diamond. Science 320, 1326–1329 (2008) . 18535240 10.1126/science.1157233

[b19] Neumann P. *et al.* Single shot readout of a single nuclear spin. Science 329, 542–544 (2010) . 20595582 10.1126/science.1189075

[b20] Robledo L. *et al.* High-fidelity projective read-out of a solid state spin quantum register. Nature 477, 574–578 (2011) . 21937989 10.1038/nature10401

[b21] Maurer P. C. *et al.* Room-temperature quantum bit memory exceeding one second. Science 336, 1283–1286 (2012) . 22679092 10.1126/science.1220513

[b22] van der Sar T. *et al.* Decoherence-protected quantum gates for a hybrid solid-state spin register. Nature 484, 82–86 (2012) . 22481361 10.1038/nature10900

[b23] Waldherr G. *et al.* Quantum error correction in a solid-state hybrid spin register. Nature 506, 204–207 (2014) . 24476818 10.1038/nature12919

[b24] Taminiau T. H., Cramer J., van der Sar T., Dobrovitski V. V. & Hanson R. Universal control and error correction in multi-qubit spin registers in diamond. Nat. Nanotechnol. 9, 171–176 (2014) . 24487650 10.1038/nnano.2014.2

[b25] Shi F. Z. *et al.* Room-temperature implementation of the deutsch-jozsa algorithm with a single electronic spin in diamond. Phys. Rev. Lett. 105, 040504 (2010) . 20867828 10.1103/PhysRevLett.105.040504

[b26] Xu X. K. *et al.* Coherence-protected quantum gate by continuous dynamical decoupling in diamond. Phys. Rev. Lett. 109, 070502 (2012) . 23006348 10.1103/PhysRevLett.109.070502

[b27] Liu G. Q., Po H. C., Du J. F., Liu R. B. & Pan X. Y. Noise resilient quantum evolution steered by dynamical decoupling. Nat. Commun. 4, 2254 (2013) . 23912335 10.1038/ncomms3254 PMC3741639

[b28] Pan X. Y., Liu G. Q., Yang L. L. & Fan H. Solid-state optimal phase-covariant quantum cloning machine. Appl. Phys. Lett. 99, 051113 (2011) .

[b29] Chang Y. C., Liu G. Q., Liu D. Q., Fan H. & Pan X. Y. Room temperature quantum cloning machine with full coherent phase control in nanodiamond. Sci. Rep. 3, 1498 (2013) . 23511233 10.1038/srep01498 PMC3603226

[b30] Hadden J. P. *et al.* Strongly enhanced photon collection from diamond defect centers under microfabricated integrated solid immersion lenses. Appl. Phys. Lett. 97, 241901 (2010) .

[b31] Smeltzer B., Childress L. & Gali A. ^13^C hyperfine interactions in the nitrogen-vacancy centre in diamond. New J. Phys. 13, 025021 (2011) .

[b32] Jacques V. *et al.* Dynamic polarization of single nuclear spins by optical pumping of nitrogen-vacancy color centers in diamond at room temperature. Phys. Rev. Lett. 102, 057403 (2009) . 19257552 10.1103/PhysRevLett.102.057403

[b33] Smeltzer B., McIntyre J. & Childress L. Robust control of individual nuclear spins in diamond. Phys. Rev. A 80, 050302(R) (2009) .

[b34] Dréau A., Spinicelli P., Maze J. R., Roch J.-F. & Jacques V. Single-shot readout of multiple nuclear spin qubits in diamond under ambient conditions. Phys. Rev. Lett. 110, 060502 (2013) . 23432227 10.1103/PhysRevLett.110.060502

[b35] Zhao N. *et al.* Sensing single remote nuclear spins. Nat. Nanotechnol. 7, 657–662 (2012) . 22941402 10.1038/nnano.2012.152

[b36] Kolkowitz S., Unterreithmeier Q. P., Bennett S. D. & Lukin M. D. Sensing distant nuclear spins with a single electron spin. Phys. Rev. Lett. 109, 137601 (2012) . 23030118 10.1103/PhysRevLett.109.137601

[b37] Taminiau T. H. *et al.* Detection and control of individual nuclear spins using a weakly coupled electron spin. Phys. Rev. Lett. 109, 137602 (2012) . 23030119 10.1103/PhysRevLett.109.137602

[b38] Zhang Y. L., Zhang Y. R., Mu L. Z. & Fan H. Criterion for remote clock synchronization with Heisenberg-scaling accuracy. Phys. Rev. A 88, 052314 (2013) .

[b39] Zhang Y. R., Jin G. R., Cao J. P., Liu W. M. & Fan H. Unbounded quantum Fisher information in two-path interferometry with finite photon number. J. Phys. A 46, 035302 (2013) .

[b40] Cramér H. Mathematical Methods of Statistics Ch. 32–34 Princeton Univ. Press (1946) .

[b41] Braunstein S. L. & Caves C. M. Statistical distance and the geometry of quantum states. Phys. Rev. Lett. 72, 3439–3443 (1994) . 10056200 10.1103/PhysRevLett.72.3439

[b42] Liu G. Q., Pan X. Y., Jiang Z. F., Zhao N. & Liu R. B. Controllable effects of quantum fluctuations on spin free-induction decay at room temperature. Sci. Rep. 2, 432 (2012) . 22666535 10.1038/srep00432 PMC3362804

[b43] Liu G. Q. *et al.* Protection of centre spin coherence by dynamic nuclear spin polarization in diamond. Nanoscale 6, 10134–10139 (2014) . 25042514 10.1039/c4nr02007c

