# Supplementary material for: Demonstration of entanglement-enhanced phase estimation in solid
Source: Nat Commun. 2015 Apr 2;6:6726. doi: 10.1038/ncomms7726 (PMC4396365; doi:10.1038/ncomms7726)
Supplement: Supplementary Information — Supplementary Figures 1-3, Supplementary Notes 1-2 and Supplementary References [file ncomms7726-s1.pdf]

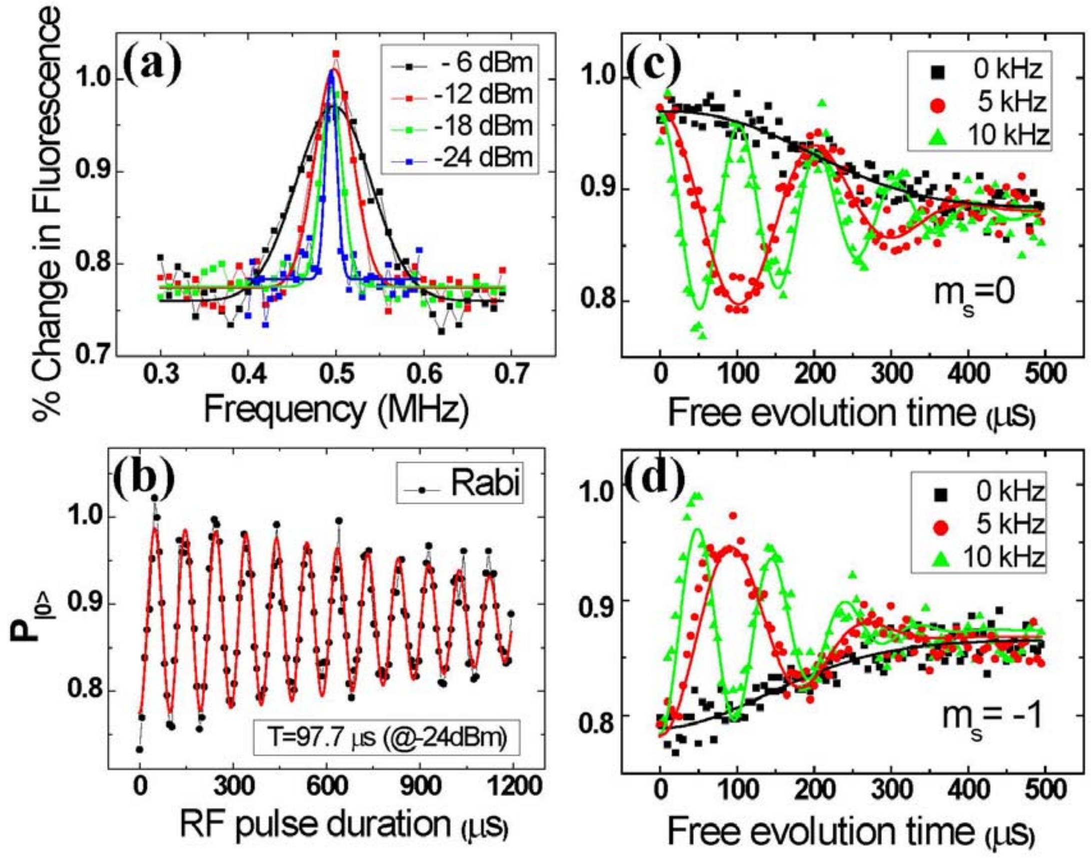

Supplementary Figure 1 | Coherence of nuclear spin. **(a)** Pulse-ODMR of ^{13}C nuclear spin with RF pulses of different driven power. The resonant frequencies are the same for all the measurement (evidence of weak driven). **(b)** Rabi oscillation of nuclear spin under weak RF driven. The measured Rabi frequency is about 10 kHz, with typical envelop decay time of $T_{1\rho}$ = 1.3 ms. **(c)** FID of nuclear spin when electron spin is at $m_S = 0$ state, with dephasing time of $T^*_{2n(ms=0)} = 270$ μs. **(d)** FID of nuclear spin when electron spin is at $m_S = -1$ state, with dephasing time of $T^*_{2n(ms=-1)} = 212$ μs. The square, circle and triangle are experiment data with detuning of 0 kHz, 5 kHz and 10 kHz, respectively. Solid lines are fitting to them.

(a)

(b)

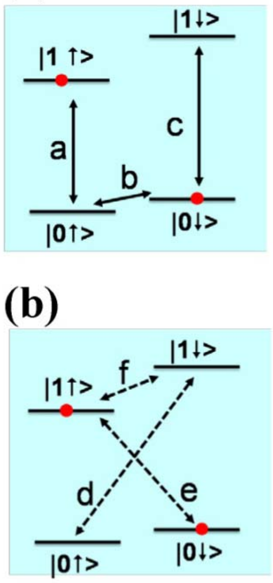

<table>
<tr><th rowspan="2">(c)</th><th rowspan="2">Elements
|0↑> ⇨1
|1↑> ⇨2
|0↓> ⇨3
|1↓> ⇨4</th><th colspan="2">Transfer Pulse</th><th colspan="2">Measurement Rabi oscillation</th></tr>
<tr><th>Transition</th><th>Duration (Phase)</th><th>Re. parts (phase)</th><th>Im. parts (phase)</th></tr>
<tr><td>a</td><td>ρ_{11} ρ_{12}
ρ_{21} ρ_{22}</td><td colspan="2">do nothing</td><td>|0↑>↔|1↑>
(0°)</td><td>|0↑>↔|1↑>
(90°)</td></tr>
<tr><td>b</td><td>ρ_{33} ρ_{34}
ρ_{43} ρ_{44}</td><td colspan="2">do nothing</td><td>|0↓>↔|1↓>
(0°)</td><td>|0↓>↔|1↓>
(90°)</td></tr>
<tr><td>c</td><td>ρ_{11} ρ_{13}
ρ_{31} ρ_{33}</td><td colspan="2">do nothing</td><td>|0↑>↔|0↓>
(0°)</td><td>|0↑>↔|0↓>
(90°)</td></tr>
<tr><td>d</td><td>ρ_{11} ρ_{14}
ρ_{41} ρ_{44}</td><td>|0↓>↔|1↓></td><td>π
(0 °)</td><td>|0↑>↔|0↓>
(0°)</td><td>|0↑>↔|0↓>
(90°)</td></tr>
<tr><td>d</td><td>ρ_{33} ρ_{32}
ρ_{23} ρ_{22}</td><td>|0↑>↔|1↑></td><td>π
(0°)</td><td>|0↑>↔|0↓>
(0°)</td><td>|0↑>↔|0↓>
(90°)</td></tr>
<tr><td rowspan="2">f</td><td rowspan="2">ρ_{44} ρ_{42}
ρ_{24} ρ_{22}</td><td>|0↓>↔|1↓></td><td>π (0 °)</td><td rowspan="2">|0↑>↔|0↓>
(0°)</td><td rowspan="2">|0↑>↔|0↓>
(90°)</td></tr>
<tr><td>|0↓>↔|1↓></td><td>π (0 °)</td></tr>
</table>

Supplementary Figure 2 | Procedure to carry out state tomography on electron-nuclear two-qubit system. (a) The three solid arrows are selected working transitions, which can be driven by MW/RF pulse directly. The real and imaginary parts of the matrix elements in each working transition are measured by RF (or MW) pulses of 0° and 90° phases, respectively. **(b)** For the other three transitions (dash arrow), one or two pulses are applied to transfer the state information to working transition (|0 ↑⟩ ⟷ |0 ↓⟩) before Rabi measurement. **(c)** Pulse duration and phase of state tomography. The diagonal elements of density matrix are measured three times, and the mean of these measurements are used.

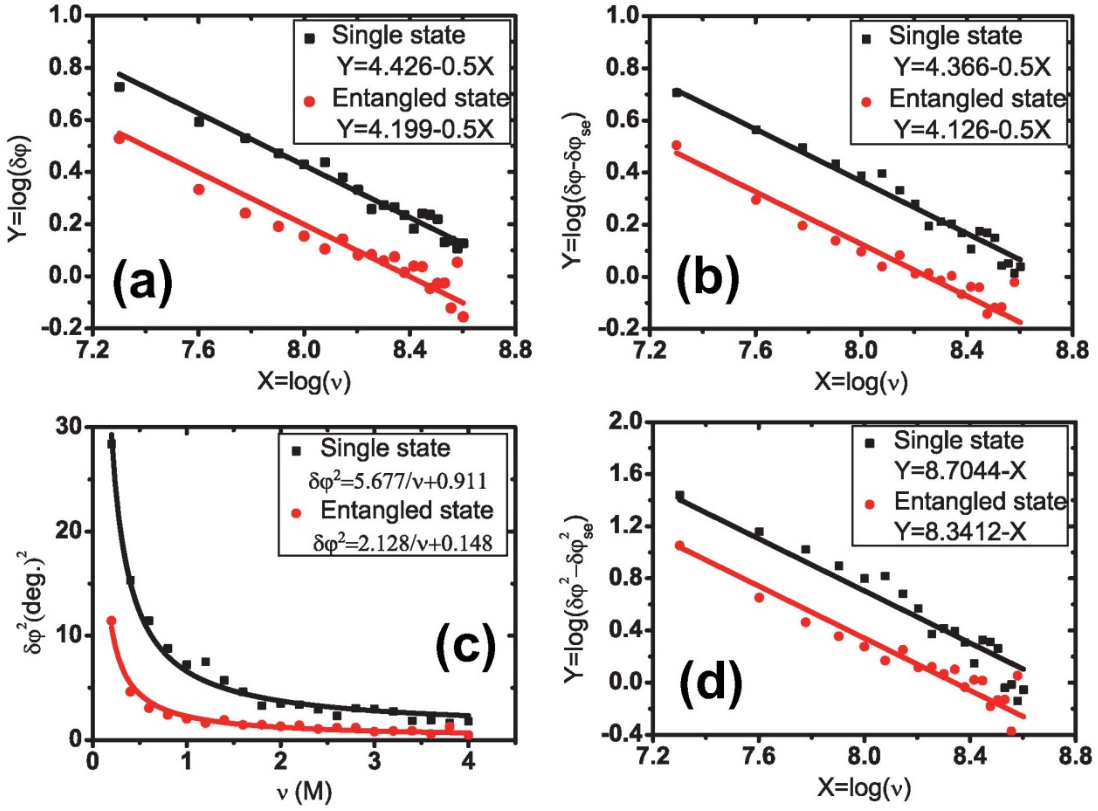

Supplementary Figure 3 | Data processing for standard deviation and variance of phase. **(a)** Linear fitting for standard deviation $\delta\varphi$ against repeat number ν in the log-log scale with adjusted R-squares 0.967 and 0.881 for single state and entangled state. **(b)** Linear fitting for standard deviation subtracted the system error $\delta\varphi_{se}$ in the log-log scale for single state and entangled state. Adjusted R-squares are 0.962 and 0.904. **(c)** The variance of phase is fitted by function $\delta\varphi^2 = a/\nu + c$ where c represents the squared system error $\delta\varphi^2$. **(d)** Linear fitting for variance subtracted the squared system error in the log-log scale. Adjusted R-squares are 0.916 and 0.893 for single state and entangled state, respectively.

Supplementary Note 1: Validity of rotating wave approximation and coherence of nuclear spin

As mentioned in the main text, the achieved Rabi frequency of nuclear spin is not small compared with the energy gap ($|0\uparrow\rangle \Leftrightarrow |0\downarrow\rangle$), thus we need to evaluate whether rotating wave approximation (RWA) still works well under this circumstance. We measure nuclear pulse-ODMR spectrum and Rabi oscillation with RF pulses of different driven powers, as shown in Supplementary Fig. 1 (a-b). The minimum Rabi frequency is only 10 kHz with a RF power of -24 dBm (at signal generator), which is much less than the energy gap of nuclear spin and RWA works well at this power. We then choose π pulse of this RF power (49 μs) to measure pulse-ODMR spectrum of nuclear spin. The resonant frequency between $|0\uparrow\rangle$ and $|0\downarrow\rangle$ transition is 495 kHz under this weak driven power. We find that the resonant frequencies are the same for all the measured RF pulses, including the one, which corresponds to a Rabi frequency of about 20% of nuclear spin energy gap. Therefore, we conclude that RWA works well for all the measurements in this experiment. This conclusion conforms with the results in Supplementary Ref.[1] and Supplementary Ref.[2], where RWA works well with a Rabi frequency less than half of the energy gap of two-level system (NV electron spin).

We then consider the coherence of nuclear spin. Supplementary Fig. 1.(c) and (d) present the FID signals of nuclear spin under ESLAC, for both $m_S = 0$ and $m_S = -1$ states of electron spin. The dephasing time of nuclear spin (T_{2n}^*=270 μs for $m_S = 0$ state and 210 μs for $m_S = -1$ state) is shorter than the result in Supplementary Ref. [3] This may be caused by the complicated spin bath of this NV center. However, similar to the case of electron spin, the dephasing time is not the limitation of nuclear manipulation duration. The Rabi envelope decay time of this nuclear spin ($T_{1\rho}$) is more than 1 ms. The half π pulse, which is used to generate the superposition state of nuclear spin, is only 5 μs and much shorter than the dephasing time. So we ignore the dephasing effect of nuclear spin in the metrology experiment.

Supplementary Note 2: Other data processing methods for standard deviation and variance

Regardless of system error, the variance of phase $\delta\varphi^2$ with a sufficiently large number of measurements ν will be approximately normally distributed as $\delta\varphi^2 \propto 1/\nu$, which is based on the classical central limit theorem. This fact can also be explained by the additive property of Fisher information and is shown in Eq. (1) in the main text. Therefore, in Fig. 4(b) we set the exponent of ν as 0.5 for standard deviation (SD) and a conclusive result is given. In Supplementary Fig. 3, we try other data processing

methods for the presentation of the entanglement-enhanced metrology. In Supplementary Fig. 3(a), we use the linear fitting in the log-log scale to analyze the SD, $\delta\varphi^2$, against ν. Ideally the slope should be 0.5 and the intercept gives the value that represents the enhancement. However, there always exists the system error which will insult the linear analysis and give an inconclusive result for large number of measurement $\nu \sim$ 1 M. We thus fix the slopes as 0.5 and give the enhancement by reading the intercepts in Supplementary Fig. 3(a). The adjusted R-square, $\overline{\mathrm{R}}^2$, (ranging from 0 to 1 with larger number indicating better fitting) for single state and entangled state are 0.967 and 0.881, respectively. In Supplementary Fig. 3(b), the system error, $\delta\varphi_{se}$, read in Supplementary Fig. 3(c) is taken into consideration and subtracted out before analyzing, which leads to better adjusted R-squares: 0.962 and 0.904 for single state and entangled state, respectively. Therefore, we conclude that the data processing method we use in the main text for SD provides a better fitting to the experimental data. We also use the function $\delta\varphi^2 = a/\nu + c$ to fit experimental data of variance, see results in Supplementary Fig. 3(c). Taking out the effect of squared system error $\delta\varphi_{\mathrm{se}}^2$, we use the linear fitting in the log-log scale and present the enhancement in Supplementary Fig. 3(d) with $\overline{\mathrm{R}}^2$ being 0.916 and 0.893 for single state and entangled state, respectively. It shows that the data processing method we use for SD in the main text is better than that for variance.

Supplementary References

1 Fuchs, G. D., Dobrovitski, V. V., Toyli, D. M., Heremans, F. J. & Awschalom, D. D. Gigahertz Dynamics of a Strongly Driven Single Quantum Spin. *Science* **326**, 1520-1522 (2009).

2 Scheuer, J. *et al.* Precise qubit control beyond the rotating wave approximation. *New J Phys* **16**, 093022 (2014).

3 Dutt, M. V. G. *et al.* Quantum register based on individual electronic and nuclear spin qubits in diamond. *Science* **316**, 1312-1316 (2007).
